# Using Genetic Algorithms in a Large Nationally Representative American Sample to Abbreviate the Multidimensional Experiential Avoidance Questionnaire

**DOI:** 10.3389/fpsyg.2016.00189

**Published:** 2016-02-24

**Authors:** Baljinder K. Sahdra, Joseph Ciarrochi, Philip Parker, Luca Scrucca

**Affiliations:** ^1^Institute for Positive Psychology and Education, Australian Catholic UniversityStrathfield, NSW, Australia; ^2^Department of Economics, University of PerugiaPerugia, Italy

**Keywords:** genetic algorithms, experiential avoidance, abbreviation, measurement, psychometrics

## Abstract

Genetic algorithms (GAs) are robust machine learning approaches for abbreviating a large set of variables into a shorter subset that maximally captures the variance in the original data. We employed a GA-based method to shorten the 62-item Multidimensional Experiential Avoidance Questionnaire (MEAQ) by half without much loss of information. Experiential avoidance or the tendency to avoid negative internal experiences is a key target of many psychological interventions and its measurement is an important issue in psychology. The 62-item MEAQ has been shown to have good psychometric properties, but its length may limit its use in most practical settings. The recently validated 15-item brief version (BEAQ) is one short alternative, but it reduces the multidimensional scale to a single dimension. We sought to shorten the 62-item MEAQ by half while maintaining fidelity to its six dimensions. In a large nationally representative sample of Americans (*N* = 7884; 52% female; Age: *M* = 47.9, *SD* = 16), we employed a GA method of scale abbreviation implemented in the R package, GAabbreviate. The GA-derived short form, MEAQ-30 with five items per subscale, performed virtually identically to the original 62-item MEAQ in terms of inter-subscales correlations, factor structure, factor correlations, and zero-order correlations and unique latent associations of the six subscales with other measures of mental distress, wellbeing and personal strivings. The two measures also showed similar distributions of means across American census regions. The MEAQ-30 provides a multidimensional assessment of experiential avoidance whilst minimizing participant burden. The study adds to the emerging literature on the utility of machine learning methods in psychometrics.

## Introduction

Recent methodological advances in scale abbreviation have demonstrated genetic algorithms (GAs) to be robust machine learning approaches to short-form construction (Yarkoni, 2010; Eisenbarth et al., 2015), which work just as well as traditional approaches (Sandy et al., 2014). Conventional methods of scale abbreviation require researchers to manually balance several recommended criteria for item selection, such as, high item-total correlations, high factor loadings, low cross-loadings, low correlated uniqueness, low missing data, high coefficient alpha, and researchers' subjective judgment of the suitability of the selected items (Marsh et al., 2005). In contrast, the GA is a completely automated, highly sophisticated optimization tool that is relatively simple to implement in freely available software, such as the Precis package in Python (see Eisenbarth et al., 2015) and the GAabbreviate package in R (Scrucca and Sahdra, 2015).

The GAs mimic Darwinian evolution principles to efficienty search for a short form of a long form measure in a fully automated manner (Holland, 1975; Scrucca, 2013). An exhaustive search of all possible shorter forms of the original long form and running their respective validation tests would be inefficient. For a long form of length *L* (e.g., 100 items), the size of the search space for any machine learning method is 2^*L*^(1.26*e*^+30^) and forms a hypercube of *L* dimensions. (A hypercube is an extension of *L*-dimensional geometric figures: a two-dimensional hypercube is a square, a three-dimensional one is a cube and so on). A GA method uses “hypercube sampling” by sampling the corners of the *L*-dimensional hypercube. It optimizes the search for a good solution—the “fittest” short form that maximally explains the variance in the data of the original long-form—by mimicking Darwinian evolution mechanisms of selection, crossover and mutation while searching through a “landscape” of the collection of all possible fitness values to find an optimal value. Selection in a GA algorithm refers to whether or not an item is selected in a particular iteration. Crossover refers to switching of two items such that the selected item in one generation is unselected in the next one and the unselected item in one generation is selected in the next one. Mutation refers to a (small) probability that an item will randomly switch status (selected or unselected) in a particular generation. The technical details of the GA method of scale abbreviation have been described elsewhere (Whitley, 1994; Yarkoni, 2010; Scrucca, 2013).

In the current study, we used a GA-based method to shorten a long measure of experiential avoidance or the tendency to avoid negative internal experiences. Measurement of experiential avoidance is a vital issue in psychology. Substantial experimental work has shown that one aspect of experiential avoidance, thought suppression, is related to paradoxical rebound effects of increased emotional and behavioral impact of thoughts (Wegner, 1989; Wegner and Erber, 1992; Abramowitz et al., 2001). Avoidance of internal states has been linked to clinical conditions such as depression, generalized anxiety, panic disorder, post traumatic distress, and many other psychopathologies (Chawla and Ostafin, 2007). Consequently, experiential avoidance is a key target of change in modern mindfulness-based psychological interventions such as acceptance and commitment therapy (Hayes et al., 2012), and dialectical behavior therapy (Linehan, 1993).

The Acceptance and Action Questionnaires (the AAQ and its revision, AAQ-II) were early attempts at assessing experiential avoidance (Mccurry et al., 2004). These measures treated experiential avoidance as a unidimensional subcomponent of a broader construct termed “psychological flexibility,” or the ability to connect with the present moment and experience thoughts and feelings openly as they arise, whilst persisting in action that is consistent with values, or changing action when the situation requires it (Ciarrochi et al., 2014). There has been some controversy around AAQ-II. It has been criticized for confounding the process or trait it is designed to measure (experiential avoidance) and the outcomes (e.g., wellbeing) of the process (Chawla and Ostafin, 2007), and for lacking discriminant validity with respect to negative emotionality (Gámez et al., 2011). However, other research suggests that experiential avoidance at the daily level can be distinguished from highly related constructs of mental distress (Kashdan et al., 2014).

The 62-item Multidimensional Experiential Avoidance Questionnaire (MEAQ) was an important attempt to improve on the AAQ-II in that it focused explicitly on key aspects of experiential avoidance without reducing them to a single dimension (Gámez et al., 2011). Specifically, the MEAQ measures the following six dimensions of experiential avoidance: behavioral avoidance (e.g., “I won't do something if I think it will make me uncomfortable”), distress aversion (e.g., “I would do anything to feel less stressed”), distraction and suppression (e.g., “When something upsetting comes up, I try very hard to stop thinking about it”), repression/denial (e.g., “I am able to turn off my emotions when I don't want to feel”), procrastination (e.g., “I tend to put off unpleasant things that need to get done”), and distress endurance (e.g., “Even when I feel uncomfortable, I don't give up working toward things I value”). Gámez et al. (2011) provided the first compelling evidence that experiential avoidance is best treated as a multidimensional construct: the six subscales were differentially related to other constructs, even beyond the effects of individual differences in negative emotionality. However, despite having good psychometric properties, the 62-item MEAQ may be too lengthy to use in many settings (Gámez et al., 2014). The recently validated 15-item Brief Questionnaire (BEAQ) reduces the administration time from about 12 to 3 min (Gámez et al., 2014), but it defeats the key purpose of the original MEAQ by collapsing across the six dimensions. Recent evidence suggests that people may have different profiles of experiential avoidance, being above average on some aspects of avoidance, and average and below average on others, and this may have important implications for practice (Ciarrochi et al., 2014).

In the current study, we employed recent methodological advances in scale abbreviation using genetic algorithms to develop a measure that was short enough to be of practical utility in most settings but not so short as to lose the multidimensional nature of experiential avoidance. We utilized a large nationally representative American sample to evaluate the extent that a reduced MEAQ was comparable to the original long form in terms of inter-subscales correlations, reliability, factor structure, and zero-order and unique associations with a variety of criterion variables. We used the following constructs relevant for convergent and discriminant validity, psychological inflexibility (Bond et al., 2011) and alexithymia (Bagby et al., 1994), both of which are expected to be related to high avoidance. Additional constructs relevant for construct validity included mental health (Goldberg et al., 1996), flourishing (Keyes, 2006), satisfaction with life (Diener et al., 1985), and personal strivings (Emmons and McAdams, 1991; Sheldon and Kasser, 2001). We expected experiential avoidance to be linked to high mental distress, low wellbeing, low life satisfaction, high importance of personal strivings but low autonomous and high controlled reasons for striving, and low progress on personal strivings. The key goal of our study was methodological: to test how well the GA-derived short form preserved the psychometric properties of the long form MEAQ. The results also allowed us to explore the relative importance of different dimensions of experiential avoidance for understanding the link between experiential avoidance and other mental distress, wellbeing and personal strivings related constructs.

## Materials and methods

### Participants and design

A nationally representative American sample (*N* = 7884; 52% female; Age: *M* = 47.9, *SD* = 16) was conducted by a professional survey company. Ethics approval was obtained from the University of Western Sydney Human Research Ethics Committee (H9798) before data collection. Participants completed an on-line anonymous survey in exchange for points they received from the survey company, which they could redeem for merchandize directly from the company. They were asked to complete the survey in a quiet place free of distractions, alone and all in one sitting. In the first part of the survey, all participants completed a measure of personal strivings, the results from which are reported elsewhere (Ciarrochi et al., 2014). For the remaining part of the survey, we utilized a planned missing data design or “matrix sampling” (Schafer, 1997; Graham et al., 2006) to keep the burden on participants to a minimum, and used multiple imputations to deal with the uncertainty related to missing data (as described in detail in Section Multiple Imputation Procedure below). Each participant received a random sample of 60 items. Each item consisted of responses from at least 21% of the sample (1655 respondents).

### Measures

#### Experiential avoidance

We used the 62-item Multidimensional Experiential Avoidance Questionnaire or MEAQ (Gámez et al., 2011). Using a scale ranging from 1 (strongly disagree) to 6 (strongly agree), participants rated their response to 62 items assessing six dimensions of avoidance: behavioral avoidance (α = 0.81; “I won't do something if I think it will make me uncomfortable”), distress aversion (α = 0.80; “I would do anything to feel less stressed”), distraction and suppression (α = 0.74; “When something upsetting comes up, I try very hard to stop thinking about it”), repression/denial (α = 0.83; “I am able to turn off my emotions when I don't want to feel”), procrastination (α = 0.77; “I tend to put off unpleasant things that need to get done”), and distress endurance (α = 0.78; “Even when I feel uncomfortable, I don't give up working toward things I value”).

#### Alexithymia

Using a scale ranging from 1 (Strongly Disagree) to 5 (Strongly Agree), participants rated their responses to two subscales of the Toronto Alexithymia Scale (Bagby et al., 1994; Landstra et al., 2013): seven items of difficulties identifying feelings (α = 0.86; e.g., “When I am upset, I don't know if I am sad, frightened, or angry”), and five items for difficulties describing feelings (α = 0.77; e.g., “It is difficult for me to find the right words for my feelings”).

#### Psychological inflexibility

We used the Acceptance and Action Questionnaire II or AAQ-II (Bond et al., 2011). The AAQ-II measures the tendency to control thoughts and feelings and ability to act in the presence of difficult thoughts or feelings. Each item is rated on a seven-point Likert scale (α = 0.87; e.g., “I worry about not being able to control my worries and feelings” and “My painful memories prevent me from having a fulfilling life”). Higher scores indicate higher psychological flexibility. The AAQ-II has been shown to have adequate test-retest reliability, discriminant, convergent, and predictive validity (Bond et al., 2011).

#### Mental health

We used a well-validated measure of personal mental health, the General Health Questionnaire (Goldberg, 1978; Goldberg et al., 1996). Participants used a four-point scale with labels such as “not at all” to “much more than usual” to respond to 12 items, all beginning with a sentence stem, “Have you recently….” Example items include: “been feeling unhappy or depressed,” “felt you couldn't overcome your difficulties.” Higher scores indicate greater psychological distress. The measure showed decent internal consistency in our sample (α = 0.87).

#### Satisfaction with life

We used a well-established measure (Diener et al., 1985), in which participant rated their responses to five items using a scale from 1 (Strongly Disagree) to 5 (Strongly Agree). Example items include: “In most ways my life is close to my ideal,” “I am satisfied with my life.” The measure showed satisfactory internal consistency (α = 0.85).

#### Flourishing

To measure the positive aspect of mental health, we used a measure consisting of 12 items measuring the following three aspects of flourishing (Keyes, 2006): emotional wellbeing (α = 0.82; e.g., “In the past month, how often have you felt happy?”); psychological wellbeing (α = 0.61; e.g., “In the past month, how often did you feel good at managing the responsibilities of your daily life?”); and social wellbeing (α = 0.72; e.g., “In the past month, how often did you feel that you belonged to a community like a social group, your school, or your neighborhood?”). Participants rated their responses using a scale with the following labels: 1 (Never), 2 (Once or twice), 3 (About once a week), 4 (Two or three times a week), 5 (Almost every day), and 6 (Every day).

#### Personal strivings

We used a measure of personal strivings (Emmons and McAdams, 1991; Sheldon and Kasser, 2001) in which participants were asked to describe four personal strivings (important goals) and respond to a series of questions about those strivings using a rating scale that ranged from 1 (Disagree Strongly) to 6 (Agree Strongly). They were asked to rate the importance of striving, the extent to which it was autonomous (three questions; e.g., “I strive for this because it makes my life more meaningful”) or controlled (two questions; e.g., “I strive for this because I would feel ashamed, guilty or anxious if I didn't strive for it”), and how much progress they had made on the striving (“In the past 10 weeks I have made progress on this striving”).

### Multiple imputation procedure

The data were missing completely at random or MCAR (Enders, 2010) because the study had missing data by design. Having an MCAR design allowed us to utilize a multiple imputation procedure to produce unbiased estimates (Little and Rubin, 1987). We used the package Amelia II (Honaker et al., 2011) in the statistical software R (R_Core_Team, 2015) to derive 25 imputations. Amelia II implements Expectation-Maximization (EM) algorithm with bootstrapping (Dempster et al., 1977; King et al., 2001; Honaker et al., 2011). In this procedure, multiple bootstrapped samples of the original incomplete data are used to draw values of the complete data. The EM algorithm draws imputed values from each set of bootstrapped parameters and automatically fills in the missing values with the imputed values. Across the imputed datasets, the observed values remain the same, but the missing values are replaced with draws from EM based predictive distribution of missing data. We confirmed the robustness of the imputation model by checking that EM convergence was normal and EM chain lengths of all 25 imputed datasets were reasonably short and consistent in length. We also used the following diagnostic functions in Amelia II to further verify the validity of the imputation model: the compare density function to check the distribution of imputed values to the distribution of observed values; and the over impute function to ensure that the observed data tended to fall within the region where it would have been imputed had it been missing instead of observed.

The imputation procedure was conducted using the entire sample to maximally utilize the EM algorithm. As recommended in machine learning applications (James et al., 2014), the sample was randomly split into a training subset (*N* = 5913; 75% of the original sample) and a testing subset (*N* = 1971; 25% of the full sample), each subset with their respective 25 imputed files. The training subset was used to run a genetic algorithm to derive a short-form of MEAQ, and only the testing subset was employed for running validation tests on the short-form.

### Genetic algorithm procedure

We employed a freely available R package, GAabbreviate (Scrucca and Sahdra, 2015), which uses the GA package (Scrucca, 2013) to efficiently implement the recently validated GA method for scale reduction (Yarkoni, 2010; Sandy et al., 2014; Eisenbarth et al., 2015). (See Appendix A in Supplementary Material for an example R code using GAabbreviate). Technical details of the GA method for scale abbreviation are described elsewhere (Yarkoni, 2010). In brief, the algorithm is designed to minimize the following fitness function:

$$\begin{array}{l} Cost=Ik+\;\overset{s}{\underset{i=1}{\sum }}w_{i}\left(1-R_{i}^{2}\right) \end{array}$$

Here, *I* represents a user-specified fixed item cost, *k* represents the number of items retained by the GA, *s* is the number of subscales in the measure, *w*_*i*_ are the weights associated with each subscale, and $R_{i}^{2}$ is the amount of variance in the *i*th subscale that can be explained by a linear combination of individual item scores. The equation above is identical to the cost function of Yarkoni (2010), except we have made the weighting of subsets more explicit in the formula. By default, *w*_*i*_ has a value of 1 in GAabbreviate, giving equal weighting to all subscales, but it is relatively easy to adjust weights if needed. GAabbreviate also allows users to constrain *k*, the number of items to be retained. Adjusting the value of *I* low or high yields longer or shorter measures respectively. When the cost of each individual item retained in each generation outweighs the cost of a loss in explained variance, the GA yields a relatively brief measure. When the cost is low, the GA yields a relatively longer measure maximizing explained variance (Yarkoni, 2010).

## Results

### GA-derived short measure

The GA procedure was run using the training sample (*N* = 5913; 75% of the full sample), as is recommended for machine learning applications (James et al., 2014). After trail runs on two imputed datasets used to fine-tune the GA parameters, the following specifications were set for separate 25 GA runs on each of the 25 imputed datasets:

- item cost = 0.05 (same as the specification in Yarkoni, 2010)
- population size = 200 (same as the specification in Yarkoni, 2010)
- maximum number of iterations in each generation = 200 (approximately double the number of iterations it took the GA to converge in trial runs)
- maximum number of items per subscale = 5 (constrained to shorten the 62-item original MEAQ measure roughly by half)
- probability of crossover between pairs of chromosomes (typically a large value) = 0.8 (the default in the GA package; Scrucca, 2013)
- probability of mutation in a parent chromosome (typically a small value) = 0.1 (the default in the GA package; Scrucca, 2013)
- elitism or the number of best fitness individuals to survive at each generation = top 5% individuals (the default in the GA package; Scrucca, 2013).

**Figure 1:**
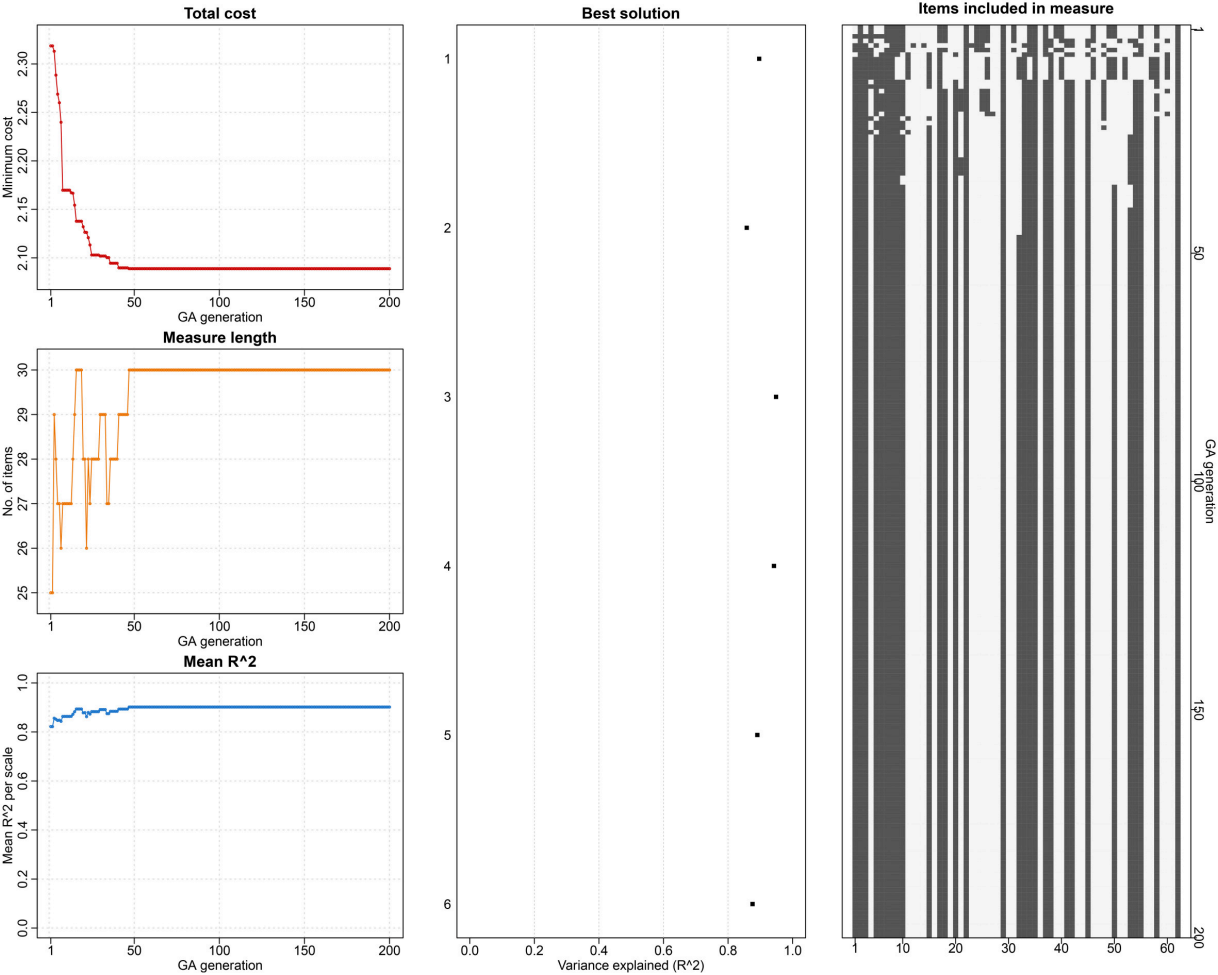
**The genetic algorithm solution for shortening the 62-item MEAQ in one of the imputed datasets**. The left panel shows the reduction in the cost, number of items retained and the mean explained variance of the selected measure across the 200 GA generations. The middle panel shows the best solution from the GA run with maximum variance explained for each of the six subscales. The right panel visually depicts the selected (black squares) and excluded (white squares) items from the first (top of the figure) to the 200^th^ (bottom of the figure) generation. This figure shows the final solution of a GA run, which can be observed from start to finish in real-time in R if the plot option in GAabbreviate is turned on.

Since the GA is a stochastic approach, the solution for each of the 25 GA runs on 25 datasets varied slightly. As an example, Figure 1 depicts the GA solution obtained for one of the imputed datasets. The left panel of the figure shows the reduction in the cost, number of items retained and the mean explained variance of the selected measure across the 200 GA generations. The middle panel shows the best solution from the GA run with maximum variance explained for each of the six subscales. The right panel visually depicts the selected (black squares) and excluded (white squares) items from the first (top of the figure) to the 200^th^ (bottom of the figure) generation. (This figure shows the final solution of a GA run, which can observed from start to finish in real-time in *R* if the plot option in GAabbreviate is turned on by adding the argument plot = TRUE to the GAabbreviate function.)

For each of the subscales of MEAQ, a list of GA-selected items from the 25 runs was created. Each item was ranked depending on the number of GA runs that selected that item (e.g., an item that was selected by 19 out of 25 GA runs received a rank of 19). The top five ranking items within each of the six subscales' list were selected to form the 30-item MEAQ (MEAQ-30 henceforth). This method allowed us to capitalize on the multiple imputation procedure accounting for missing-data uncertainty (Rubin, 1987; Schafer, 1997). Table 1 contains the items of the MEAQ-30. Further validation tests (reported below) on the MEAQ-30 employed only the testing sample's (*N* = 1971) respective 25 imputed datasets.

**Table 1:** **The 30-item Multidimensional Experiential Avoidance Questionnaire derived using a genetic algorithm for scale reduction**.

**BEHAVIORAL AVOIDANCE**
• I won't do something if I think it will make me uncomfortable • I avoid activities if there is even a small possibility of getting hurt • If I am starting to feel trapped, I leave the situation immediately • If I am in a slightly uncomfortable situation, I try to leave right away • I avoid situations if there is a chance that I'll feel nervous
**DISTRESS AVERSION**
• If I could magically remove all of my painful memories, I would • Happiness means never feeling any pain or disappointment • One of my big goals is to be free from painful emotions • I'd do anything to feel less stressed • I would give up a lot not to feel bad
**PROCRASTINATION**
• I tend to put off unpleasant things that need to get done • When I have something important to do I find myself doing a lot of other things instead • I try to put off unpleasant tasks for as long as possible • I won't do something until I absolutely have to • I try to deal with problems right away (reversed item)
**DISTRACTION AND SUPPRESSION**
• When negative thoughts come up, I try to fill my head with something else • When upsetting memories come up, I try to focus on other things • I work hard to keep out upsetting feelings • When unpleasant memories come to me, I try to put them out of my mind • When a negative thought comes up, I immediately try to think of something else
**REPRESSION/DENIAL**
• Others have told me that I suppress my feelings • It's hard for me to know what I'm feeling • It takes me awhile to realize when I'm feeling bad • I feel disconnected from my emotions • People have told me that I'm not aware of my problems
**DISTRESS ENDURANCE**
• Even when I feel uncomfortable, I don't give up working toward things I value • When I am hurting, I still do what needs to be done • I don't let pain and discomfort stop me from getting what I want • I don't let gloomy thoughts stop me from doing what I want • When working on something important, I won't quit even if things get difficult

### Subscales intercorrelations

**Figure 2:**
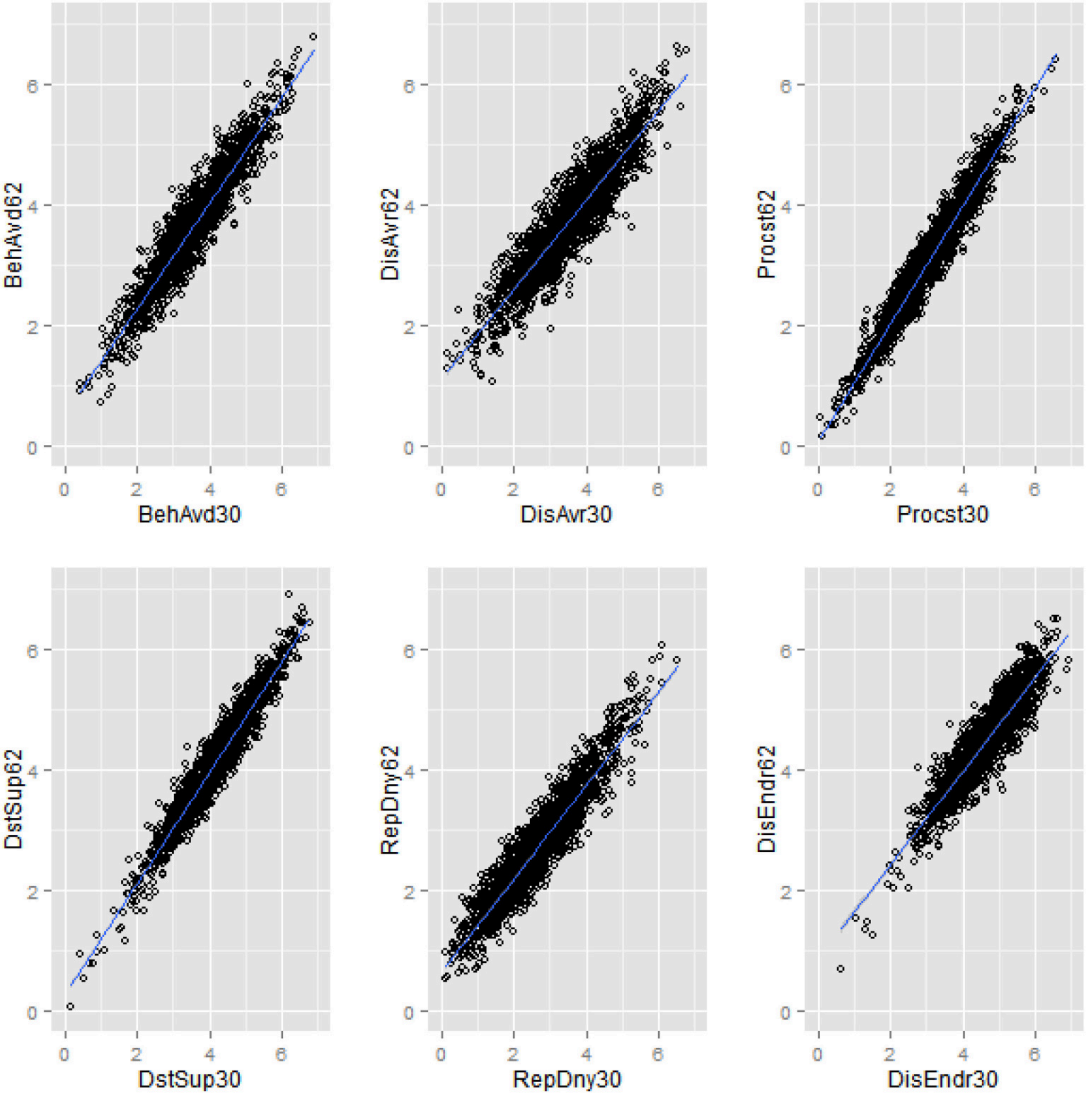
**Scatterplots displaying convergent correlations of each of the six subscales of the 62-item MEAQ with the respective subscales of the 30-item MEAQ in one of the imputed datasets**. The short MEAQ measure (plotted on the x-axis) has a high correlation with the original long form (plotted on the y-axis) for each of the subscales. BehAvd, Behavioral avoidance; DisAvr, Distress aversion; Procst, Procrastination; DstSup, Distraction and suppression; RepDny, Repression/denial; DisEndr, Distress endurance.

The pattern of intercorrelations of the subscales of the GA-derived MEAQ-30 (above diagonal in Table 2A) was very similar to the respective subscales' intercorrelations of the 62-item MEAQ (below diagonal of Table 2A). Further, the extent to which the subscales were intercorrelated in the long-form MEAQ was comparable to the correlations of the subscales of the long form with the subscales of the short form (compare the correlations below diagonal in Table 2A with the correlations in the rows of Table 2B). Similarly, the extent to which the subscales were intercorrelated in the MEAQ-30 was similar to the correlations of the subscales of MEAQ-30 with the subscales of the original 62-item MEAQ (compare the above diagonal correlations in Table 2A with the columns in Table 2B). In addition, the high correlations (all above 90) in the diagonal of Table 2B show high convergence for each of the subscale of the two MEAQ measures. Figure 2 visually depicts the high convergence in the two versions of MEAQ in scatterplots built using data from a randomly selected imputed file. The short MEAQ measure (plotted on the x-axis) has a high correlation with the original long form (plotted on the y-axis) for each of the subscales. Since the correlations of the subscales of the original MEAQ and MEAQ-30 can be artifactually inflated due to the common items both measures share in each of the subscales, it is important to do additional tests (reported below) to see if the MEAQ-30 performs similarly to the original MEAQ in terms of factor structure and construct validity.

**Table 2:** **Intercorrelations (A) between the six subscales of the 62-item MEAQ (below diagonal) and the MEAQ-30 (above diagonal), and (B) between the six subscales of the 62-item MEAQ and 30-item MEAQ**.

**(A)**						
	**BehAvd**	**DisAvr**	**Procst**	**DstSup**	**RepDny**	**DisEndr**
BehAvd		0.542^**^	0.455^**^	0.390^**^	0.372^**^	−0.110^**^
DisAvr	0.618^**^		0.336^**^	0.345^**^	0.439^**^	−0.115^**^
Procst	0.480^**^	0.352^**^		0.056	0.481^**^	−0.356^**^
DstSup	0.479^**^	0.468^**^	0.136^**^		0.165^**^	0.354^**^
RepDny	0.419^**^	0.438^**^	0.529^**^	0.224^**^		−0.202^**^
DisEndr	−0.093^**^	−0.023	−0.247^**^	0.282^**^	−0.100^**^	
**(B)**						
	**BehAvd30**	**DisAvr30**	**Procst30**	**DstSup30**	**RepDny30**	**DisEndr30**
BehAvd62	0.946^**^	0.575^**^	0.477^**^	0.418^**^	0.396^**^	−0.095^**^
DisAvr62	0.580^**^	0.927^**^	0.355^**^	0.424^**^	0.420^**^	−0.064^*^
Procst62	0.460^**^	0.339^**^	0.972^**^	0.061	0.497^**^	−0.341^**^
DstSup62	0.443^**^	0.393^**^	0.127^**^	0.962^**^	0.233^**^	0.311^**^
RepDny62	0.397^**^	0.464^**^	0.511^**^	0.161^**^	0.948^**^	−0.224^**^
DisEndr62	−0.116^**^	−0.070^*^	−0.268^**^	0.306^**^	−0.088^**^	0.904^**^

*Note that the pattern of inter-correlations is very similar across the two tables. (These are zero-order correlations pooled across 25 imputed files of the testing sample, N = 1971). BehAvd, Behavioral avoidance; DisAvr, Distress aversion; Procst, Procrastination; DstSup, Distraction and suppression; RepDny, Repression/denial; DisEndr, Distress endurance. The suffixes 62 and 30 represent the 62-item MEAQ and MEAQ-30 respectively*.

* *p < 0.05*,

** *p < 0.001*.

### Factor structure and reliability

A confirmatory factor analyses (CFA) of the original 62-item MEAQ with six factors yielded a good fit, $\chi_{\left(1814\right)}^{2}=2036.11$, *p* < 0.001, CFI = 0.96, TLI = 0.95, RMSEA = 0.01, 95% CI [0.006 0.01]. A six-factor CFA of the GA-derived MEAQ-30 also yielded as good, if not better, fit, $\chi_{\left(390\right)}^{2}=469.68$, *p* < 0.001, CFI = 0.97, TLI = 0.97, RMSEA = 0.01, 95% CI [0.006 0.01] (For interested readers, Appendix B in Supplementary Material contains the fit of a short measure derived using a conventional manual method of item selection). As with the subscales intercorrelations, the 62-item MEAQ and the GA-derived MEAQ-30 showed virtually identical patterns of factor correlations (included in Table 3): The mean of absolute values of the differences in the inter-subscale zero-order correlations (in Table 2A) was 0.06, and the mean of the differences in inter-factor correlations (in Table 3) was 0.05, both numbers being very small. Furthermore, as reported in Table 4, the alpha coefficients and average inter-item correlations of all the subscales of the 62-item and 30-item MEAQ measure were comparable. In short, the two measures yielded similar factor structure and reliability.

**Table 3:** **Correlations between the six factors from the confirmatory factor analyses of the 62-item and the 30-item MEAQ measures**.

	**BehAvd**	**DisAvr**	**Procst**	**DstSup**	**RepDny**	**DsEndr**
**THE 62-ITEM MEAQ**						
BehAvd	1.00					
DisAvr	0.71	1.00				
Procst	0.58	0.43	1.00			
DstSup	0.53	0.51	0.18	1.00		
RepDny	0.49	0.53	0.62	0.20	1.00	
DisEndr	−0.12	−0.07	−0.32	0.38	−0.30	1.00
**THE MEAQ-30**						
BehAvd	1.00					
DisAvr	0.71	1.00				
Procst	0.63	0.45	1.00			
DstSup	0.45	0.41	0.11	1.00		
RepDny	0.49	0.54	0.59	0.14	1.00	
DisEndr	−0.17	−0.14	−0.37	0.46	−0.31	1.00

*BehAvd, Behavioral avoidance; DisAvr, Distress aversion; Procst, Procrastination; DstSup, Distraction and suppression; RepDny, Repression/denial; DisEndr, Distress endurance*.

**Table 4:** **The Cronbach's alpha coefficients and average inter-item correlations of each of the subscales of the 62-item and 30-item MEAQ measures**.

	**BehAvd**	**DisAvr**	**Procst**	**DstSup**	**RepDny**	**DisEndr**
**THE 62-ITEM MEAQ**						
Cronbach's alpha	0.81	0.80	0.77	0.74	0.83	0.78
Average inter-item correlation	0.39	0.31	0.43	0.40	0.34	0.31
**THE MEAQ-30**						
Cronbach's alpha	0.78	0.76	0.78	0.78	0.79	0.80
Average inter-item correlation	0.39	0.38	0.41	0.43	0.43	0.44

*BehAvd, Behavioral avoidance; DisAvr, Distress aversion; Procst, Procrastination; DstSup, Distraction and suppression; RepDny, Repression/denial; DisEndr, Distress endurance*.

### Construct validity

Table 5 reports zero-order correlations of each of the six dimensions of the 62-item MEAQ (Table 5A) and the respective dimensions of the MEAQ-30 (Table 5B) with the criterion variables. The variables relevant for convergent and discriminant validity, alexithymia and psychological flexibility, show similar pattern of correlations across Tables 5A,B. Other variables relevant for construct validity, mental distress, satisfaction with life, the three aspects of wellbeing and the four aspects of strivings, also show similar correlations across the two forms of MEAQ. The mean of absolute values of the differences in the zero-order correlations of each of the two MEAQ measures with all other measures was only 0.03, suggesting that the MEAQ-30 performed virtually identically to the original MEAQ in terms of the associations of the six subscales with other variables.

**Table 5:** **Coefficient alphas and zero-order correlations of (A) each of the six dimensions of the 62-item with other constructs, and (B) each of the subscales of the MEAQ-30 with other constructs**.

**(A)**						
	**BehAvd62**	**DisAvr62**	**Procst62**	**DstSup62**	**RepDny62**	**DisEndr62**
α	0.81	0.80	0.77	0.74	0.83	0.78
identifyTAS	0.444^**^	0.505^**^	0.515^**^	0.168^**^	0.757^**^	−0.192^**^
describeTAS	0.375^**^	0.350^**^	0.462^**^	0.110^**^	0.701^**^	−0.234^**^
AAQ2	0.499^**^	0.596^**^	0.563^**^	0.152^**^	0.682^**^	−0.217^**^
GHQ	0.357^**^	0.410^**^	0.438^**^	0.070^*^	0.519^**^	−0.184^**^
SWL	−0.053	−0.128^**^	−0.193^**^	0.190^**^	−0.073^*^	0.423^**^
emoWB	−0.075^*^	−0.146^**^	−0.278^**^	0.232^**^	−0.227^**^	0.513^**^
psychWB	−0.052	−0.034	−0.224^**^	0.265^**^	−0.148^**^	0.577^**^
socialWB	0.048	0.063^*^	−0.031	0.238^**^	0.095^**^	0.384^**^
strvImportance	0.011	0.085^**^	−0.169^**^	0.187^**^	−0.243^**^	0.331^**^
strvAutonomous	0.090^**^	0.184^**^	−0.105^**^	0.259^**^	−0.084^**^	0.379^**^
strvControlled	0.333^**^	0.380^**^	0.314^**^	0.157^**^	0.447^**^	−0.045
strvProgress	0.019	0.062	−0.142^**^	0.175^**^	0.023	0.329^**^
**(B)**						
	**BehAvd30**	**DisAvr30**	**Procst30**	**DstSup30**	**RepDny30**	**DisEndr30**
α	0.78	0.76	0.78	0.78	0.79	0.80
identifyTAS	0.430^**^	0.524^**^	0.497^**^	0.098^**^	0.742^**^	−0.321^**^
describeTAS	0.356^**^	0.348^**^	0.454^**^	0.054	0.704^**^	−0.304^**^
AAQ2	0.485^**^	0.609^**^	0.552^**^	0.061^*^	0.666^**^	−0.363^**^
GHQ	0.338^**^	0.432^**^	0.421^**^	0.004	0.499^**^	−0.288^**^
SWL	−0.052	−0.170^**^	−0.211^**^	0.231^**^	−0.065	0.447^**^
emoWB	−0.081^*^	−0.200^**^	−0.296^**^	0.283^**^	−0.211^**^	0.561^**^
psychWB	−0.072	−0.075^*^	−0.234^**^	0.306^**^	−0.144^**^	0.581^**^
socialWB	0.046	0.041	−0.054	0.256^**^	0.102^**^	0.370^**^
strvImportance	0.012	0.040	−0.167^**^	0.207^**^	−0.220^**^	0.327^**^
strvAutonomous	0.079^*^	0.129^**^	−0.114^**^	0.273^**^	−0.087^**^	0.342^**^
strvControlled	0.319^**^	0.358^**^	0.312^**^	0.120^**^	0.424^**^	−0.110^*^
strvProgress	0.003	0.049	−0.142^**^	0.194^**^	0.027	0.300^**^

*Compare each cell across the two tables to note the similarity of the estimates*.

* *p < 0.05*,

** *p < 0.001*.

One limitation of zero-order correlations is that they do not show unique contribution of each experiential avoidance dimension, controlling for other dimensions, in explaining the variance in the criterion variables. To examine such unique relations as accurately as possible, that is, by removing as much measurement error as possible in each MEAQ measure, we ran structural equation models. We used the R packages lavaan (Rosseel, 2012) and semTools (Pornprasertmanit et al., 2013) to run these models using the 25 imputed dataset of the validation sample. See Table 6 for goodness of fit indices, and Tables 7–9 for standardized regression coefficients and *R*^2^ from these models. The mean of absolute values of the differences in regression coefficients of the two MEAQ measures predicting the outcomes was only 0.02, suggesting that the MEAQ-30 performed almost identically to the 62-item MEAQ. Further, the *R*^2^ values from these models show that the MEAQ-30, compared to the original MEAQ, consistently explained slightly more or similar degree of variance in the outcomes.

**Table 6:** **Summary of goodness of fit for models using the 62-item MEAQ to predict other constructs and models using the 30-item MEAQ to predict the same constructs**.

	**χ^2^**	***df***	**CFI**	**TLI**	**RMSEA [95% CI]**
**PREDICTING TWO ASPECTS OF ALEXITHYMIA**					
Model using the 62-item MEAQ	2702.96	2599	0.98	0.98	0.005 [0.000 0.007]
Model using MEAQ-30	900.48	791	0.97	0.97	0.008 [0.005 0.011]
**PREDICTING PSYCHOLOGICAL FLEXIBILITY**					
Model using the 62-item MEAQ	2389.98	2256	0.98	0.98	0.005 [0.002 0.008]
Model using MEAQ-30	698.61	608	0.98	0.98	0.009 [0.005 0.012]
**PREDICTING MENTAL DISTRESS**					
Model using the 62-item MEAQ	2671.14	2606	0.99	0.99	0.004 [0.000 0.006]
Model using MEAQ-30	408.73	384	0.99	0.99	0.006 [0.000 0.010]
**PREDICTING LIFE SATISFACTION**					
Model using the 62-item MEAQ	2224.61	2123	0.98	0.98	0.005 [0.000 0.007]
Model using MEAQ-30	591.80	539	0.98	0.98	0.007 [0.000 0.010]
**PREDICTING THREE ASPECTS OF FLOURISHING**					
Model using the 62-item MEAQ	2649.84	2591	0.99	0.99	0.003 [0.000 0.006]
Model using MEAQ-30	923.32	783	0.96	0.96	0.010 [0.007 0.012]
**PREDICTING FOUR ASPECTS OF STRIVING**					
Model using the 62-item MEAQ	2420.91	2234	0.97	0.97	0.007 [0.004 0.008]
Model using MEAQ-30	704.01	586	0.97	0.96	0.010 [0.007 0.013]

**Table 7:** **Regression coefficients and *R*^2^ from structural equation models showing unique relationships of the subscales of the 62-item and the 30-item MEAQ measures with alexithymia (difficulty identifying and describing internal states), psychological flexibility, and mental distress**.

	**Standardized estimates of unique associations**						**Percent variance explained**	
	**MEAQ**			**MEAQ-30**			**MEAQ**	**MEAQ-30**
	**Estimate**	***SE***	***p***	**Estimate**	***SE***	***p***	***R^2^***	***R^2^***
**Alexithymia: difficulty identifying feelings**							0.88	0.90
BehAvd	0.00	0.06	1.00	0.07	0.09	0.39		
DisAvr	0.19	0.04	0.00	0.18	0.06	0.01		
Procst	−0.01	0.04	0.89	−0.04	0.05	0.49		
DstSup	−0.07	0.06	0.15	−0.07	0.05	0.19		
RepDny	0.81	0.05	0.00	0.80	0.07	0.00		
DisEndr	−0.07	0.07	0.12	−0.11	0.05	0.01		
**Alexithymia: difficulty describing feelings**							0.76	0.86
BehAvd	0.13	0.08	0.07	0.25	0.14	0.04		
DisAvr	−0.06	0.05	0.26	−0.16	0.08	0.08		
Procst	−0.03	0.04	0.57	−0.07	0.06	0.28		
DstSup	−0.02	0.07	0.74	−0.02	0.08	0.81		
RepDny	0.85	0.05	0.00	0.88	0.08	0.00		
DisEndr	−0.09	0.06	0.03	−0.11	0.07	0.07		
**Psychological (in)flexibility**							0.81	0.84
BehAvd	0.00	0.08	0.97	0.06	0.14	0.48		
DisAvr	0.49	0.07	0.00	0.49	0.07	0.00		
Procst	0.13	0.05	0.00	0.12	0.07	0.04		
DstSup	−0.23	0.08	0.00	−0.25	0.08	0.00		
RepDny	0.50	0.04	0.00	0.45	0.07	0.00		
DisEndr	−0.05	0.08	0.13	−0.06	0.08	0.21		
**Mental distress**							0.60	0.62
BehAvd	0.01	0.03	0.89	0.03	0.04	0.76		
DisAvr	0.35	0.02	0.00	0.39	0.02	0.00		
Procst	0.14	0.02	0.01	0.12	0.02	0.07		
DstSup	−0.23	0.03	0.00	−0.25	0.03	0.00		
RepDny	0.46	0.02	0.00	0.42	0.02	0.00		
DisEndr	−0.05	0.03	0.30	−0.05	0.02	0.45		

*BehAvd, Behavioral avoidance; DisAvr, Distress aversion; Procst, Procrastination; DstSup, Distraction and suppression; RepDny, Repression/denial; DisEndr, Distress endurance*.

**Table 8:** **Regression coefficients and *R*^2^ from structural equation models showing unique relationships of the subscales of the 62-item MEAQ and the MEAQ-30 with the three aspects of flourishing, and satisfaction with life**.

	**Standardized estimates of unique associations**						**Percent variance explained**	
	**MEAQ**			**MEAQ-30**			**MEAQ**	**MEAQ-30**
	**Estimate**	***SE***	***p***	**Estimate**	***SE***	***p***	***R^2^***	***R^2^***
**Emotional wellbeing**							0.52	0.56
BehAvd	0.14	0.09	0.07	0.19	0.15	0.12		
DisAvr	−0.31	0.06	0.00	−0.33	0.09	0.00		
Procst	−0.08	0.05	0.22	−0.07	0.08	0.42		
DstSup	0.21	0.10	0.01	0.18	0.10	0.06		
RepDny	−0.03	0.05	0.53	−0.03	0.06	0.64		
DisEndr	0.55	0.14	0.00	0.57	0.11	0.00		
**Psychological wellbeing**							0.67	0.70
BehAvd	0.08	0.08	0.35	0.02	0.15	0.88		
DisAvr	−0.11	0.04	0.06	−0.08	0.07	0.45		
Procst	−0.05	0.04	0.32	0.02	0.07	0.80		
DstSup	0.08	0.08	0.33	0.05	0.11	0.67		
RepDny	−0.11	0.05	0.09	−0.12	0.07	0.15		
DisEndr	0.72	0.13	0.00	0.77	0.10	0.00		
**Social wellbeing**							0.33	0.34
BehAvd	−0.06	0.12	0.53	−0.06	0.20	0.66		
DisAvr	−0.12	0.08	0.16	−0.07	0.10	0.51		
Procst	0.01	0.06	0.92	0.06	0.10	0.55		
DstSup	0.17	0.10	0.03	0.11	0.11	0.24		
RepDny	0.24	0.08	0.00	0.21	0.10	0.03		
DisEndr	0.52	0.13	0.00	0.55	0.11	0.00		
**Satisfaction with life**							0.32	0.36
BehAvd	0.13	0.07	0.05	0.19	0.12	0.07		
DisAvr	−0.36	0.06	0.00	−0.40	0.06	0.00		
Procst	−0.13	0.05	0.05	−0.12	0.07	0.13		
DstSup	0.19	0.08	0.01	0.17	0.09	0.06		
RepDny	0.17	0.05	0.01	0.19	0.06	0.00		
DisEndr	0.43	0.11	0.00	0.44	0.09	0.00		

*BehAvd, Behavioral avoidance; DisAvr, Distress aversion; Procst, Procrastination; DstSup, Distraction and suppression; RepDny, Repression/denial; DisEndr, Distress endurance*.

**Table 9:** **Regression coefficients and *R*^2^ from structural equation models showing unique relationships of the subscales of the 62-item MEAQ and the MEAQ-30 with the four aspects of the first reported personal striving**.

	**Standardized estimates of unique associations**						**Percent variance explained**	
	**MEAQ**			**MEAQ-30**			**MEAQ**	**MEAQ-30**
	**Estimate**	***SE***	***p***	**Estimate**	***SE***	***p***	***R^2^***	***R^2^***
**Striving Importance**							0.12	0.11
BehAvd	−0.04	0.07	0.65	−0.08	0.11	0.44		
DisAvr	0.16	0.05	0.01	0.19	0.06	0.02		
Procst	0.10	0.05	0.11	0.12	0.06	0.11		
DstSup	0.01	0.09	0.93	0.03	0.08	0.71		
RepDny	−0.26	0.04	0.00	−0.29	0.06	0.00		
DisEndr	0.24	0.08	0.00	0.21	0.07	0.00		
**Striving Autonomously**							0.23	0.20
BehAvd	−0.03	0.08	0.70	−0.07	0.10	0.48		
DisAvr	0.19	0.05	0.01	0.22	0.07	0.02		
Procst	0.08	0.05	0.23	0.11	0.06	0.19		
DstSup	0.05	0.07	0.52	0.08	0.07	0.34		
RepDny	−0.10	0.04	0.06	−0.15	0.06	0.05		
DisEndr	0.43	0.08	0.00	0.37	0.07	0.00		
**Striving for Controlled Reasons**							0.38	0.36
BehAvd	0.07	0.11	0.39	0.10	0.18	0.39		
DisAvr	0.19	0.09	0.01	0.14	0.12	0.24		
Procst	0.01	0.07	0.85	0.05	0.10	0.55		
DstSup	−0.05	0.11	0.48	−0.04	0.10	0.63		
RepDny	0.46	0.07	0.00	0.43	0.10	0.00		
DisEndr	0.01	0.12	0.87	0.02	0.10	0.80		
**Striving Progress**							0.09	0.08
BehAvd	0.01	0.10	0.89	−0.04	0.16	0.64		
DisAvr	−0.05	0.07	0.38	0.00	0.09	0.98		
Procst	−0.11	0.07	0.06	−0.08	0.09	0.31		
DstSup	0.08	0.10	0.19	0.08	0.09	0.18		
RepDny	0.14	0.06	0.00	0.12	0.07	0.03		
DisEndr	0.24	0.11	0.00	0.21	0.08	0.00		

*BehAvd, Behavioral avoidance; DisAvr, Distress aversion; Procst, Procrastination; DstSup, Distraction and suppression; RepDny, Repression/denial; DisEndr, Distress endurance*.

### Distribution of means in USA

Finally, using all available data, we explored the distribution of mean levels of the six dimensions of experiential avoidance in the USA to see if the means of the 30-item measure were similar to the 62-item measure across the six census regions. Using participants' IP addresses, we obtained geographical information using the ip2coordinates application programming interface of Data Science Toolkit (Warden, 2011). Accurate geographical data were available from 6429 participants. Table 10 reports sample sizes in each of the American census regions, along with means and standard deviations of the six dimensions of experiential avoidance in the regions for both the 62-item and 30-item MEAQ measures. The mean of absolute values of the differences in the means of the two MEAQ measures reported in Table 10 was 0.09, a negligible difference on average.

**Table 10:** **Means and standard deviations of each of the six subscales of the 62-item and 30-item MEAQ measures in the five census regions of United States**.

	**MEAQ**		**MEAQ-30**	
	**Mean**	***SD***	**Mean**	***SD***
**MIDWEST (*N* = 1700)**				
BehAvd	3.65	0.77	3.57	0.84
DisAvr	3.68	0.74	3.49	0.88
Procst	3.15	0.87	3.13	0.86
DstSup	4.06	0.73	4.11	0.76
RepDny	2.68	0.76	2.66	0.89
DisEndr	4.45	0.63	4.63	0.75
**NORTHEAST (*N* = 1304)**				
BehAvd	3.62	0.78	3.53	0.84
DisAvr	3.70	0.77	3.51	0.91
Procst	3.17	0.90	3.16	0.89
DstSup	4.01	0.76	4.06	0.79
RepDny	2.67	0.80	2.63	0.95
DisEndr	4.43	0.68	4.59	0.80
**PACIFIC (*N* = 28)**				
BehAvd	3.77	0.69	3.72	0.83
DisAvr	3.80	0.74	3.49	0.81
Procst	3.51	0.93	3.52	0.95
DstSup	4.14	0.66	4.17	0.73
RepDny	2.67	0.49	2.66	0.60
DisEndr	4.44	0.56	4.55	0.73
**SOUTH (*N* = 2102)**				
BehAvd	3.70	0.80	3.63	0.85
DisAvr	3.72	0.78	3.54	0.92
Procst	3.13	0.89	3.12	0.88
DstSup	4.14	0.76	4.20	0.79
RepDny	2.68	0.82	2.64	0.96
DisEndr	4.52	0.63	4.70	0.76
**WEST (*N* = 1295)**				
BehAvd	3.65	0.78	3.57	0.85
DisAvr	3.69	0.78	3.49	0.92
Procst	3.17	0.87	3.16	0.86
DstSup	4.05	0.77	4.11	0.79
RepDny	2.67	0.77	2.63	0.90
DisEndr	4.50	0.64	4.66	0.77

## Discussion

The key goal of our study was methodological—to use recent machine learning advances in psychometrics employing genetic algorithms to shorten the 62-item MEAQ by half whilst preserving its psychometric properties, including its multidimensional structure. In a large nationally representative American sample with a wide age range, the GA-derived short measure, MEAQ-30 performed virtually identically to the original MEAQ in terms of intercorrelations of the subscales, factor structure and reliability estimates, average levels of the subscales in the five census regions of USA, and zero-order correlations and unique latent associations of the six dimensions of experiential avoidance with other measures of personal strivings, mental health and wellbeing. The MEAQ-30 maintains fidelity to the six sub-dimensions of experiential avoidance measured by the original 62-item but takes about half as long to complete. The results make an important contribution to the nascent field of machine learning approaches in psychometrics by demonstrating the validity of GAs as relatively easy to use optimization tools for scale abbreviation.

The GA method is highly efficient and does not take much computing time. GAabbreviate can use parallel processing to speed up computation. Also, turning the plot argument off while running the GA procedure (the default in GAabbreviate) speeds up the run time while allowing users to plot the data after a solution has been obtained. To give concrete figures of the computing time involved, we recorded the computing time for a GA run on one of the imputed subsets of our testing sample (*N* = 1971, 62 items of MEAQ, 6 subscales) using the GA parameters reported in the paper. These computations were run on a MacBook Pro with 2.6 GHz Intel Core i7 processor and 16 GB RAM. A single iteration of fitness evaluation took 0.40 s. A complete run of 200 iterations took 49.36 s without parallel processing and 25.87 s. (Parallel processing can be switched on by adding the argument parallel=TRUE to the GAabbreviate function) The computing times reported above are averages of 10 replications. In sum, the R package GAabbrevaite allows a highly efficient implementation of the GA method without much computational demand.

Our results also make a substantive contribution to psychology by highlighting the value of treating experiential avoidance as a multidimensional construct. As initial evidence of the distinctiveness of the six dimensions of experiential avoidance, the intercorrelations of the subscales of the MEAQ-30 (and the 62-item MEAQ) ranged from negligible to moderate in size, which is consistent with previous research using the 62-item MEAQ (Gámez et al., 2011; Ciarrochi et al., 2014). As further evidence, the correlations of each MEAQ measure with other variables were also different across the experiential avoidance dimensions. Perhaps the most compelling evidence comes from the unique latent relations of the six subscales with other variables, which paint a complex picture of how different experiential avoidance dimensions had different unique relations with other variables. For instance, distress aversion, but not distress endurance, was uniquely associated with difficulty identifying feelings (one aspect of alexithymia). However, distress endurance, but not distress aversion, was important for explaining unique variance in difficulty describing feelings (the other aspect of alexithymia). As another example, behavioral avoidance and distress endurance were not uniquely associated with mental distress, but they did explain unique variance in satisfaction with life. Distress endurance seemed to be the most important of all six experiential avoidance dimensions in accounting for unique variance in positive outcomes of wellbeing and life satisfaction, but it did little in explaining the variance of the negative outcomes of alexithymia, psychological (in)flexibility, and general mental distress. Repression/denial appeared to be the most important experiential avoidance dimension in accounting for the variance in the negative outcomes, but other dimensions of experiential avoidance also mattered to varying degrees, depending on the criterion variable.

Research on the multidimensional nature of experiential avoidance is in its infancy. We hesitate to make strong conclusions regarding specific aspects of experiential avoidance based on our data because we did not have specific a priori predictions about unique relationships of different experiential avoidance dimensions with mental health and wellbeing related outcomes. Our primary goal was to use a GA-based method to shorten the 62-item MEAQ without much loss of information. To that end, the MEAQ-30 captured the complex pattern of the associations of the six experiential avoidance dimensions with other variables virtually identically to the original 62-item MEAQ. We hope that our results would guide hypothesis for future research to advance a more nuanced understanding of the different dimensions of experiential avoidance.

Although comparison of the experiential avoidance levels in US census regions was not the main goal of this study, our data suggest that the mean levels of the six aspects of experiential avoidance were comparable across the census regions. The conclusions of the present study are limited to the English speaking Americans. Future studies should examine the psychometric properties of the MEAQ-30 in other languages and cultures, and clinical populations, and its test-re-test reliability and stability of the factor structure over time. Nevertheless, the MEAQ-30 may help researchers and practitioners evaluate the different experiential avoidance dimensions in half the time it takes to administer the original MEAQ.

Lengthy questionnaires tend to burden participants, which can result in poor data quality and high attrition rate (Cook et al., 2000). The 62-item MEAQ takes about 12 min to complete and can add to participant burden in a long battery. The 15-item alternative, the BEAQ, is problematic because it collapses across the multidimensional nature of experiential avoidance. One of the dangers of using excessively short versions of well-constructed longer measures is that they can increase both the Type 1 and Type 2 error rates by underestimating or overestimating the relations of the construct with other measures (Smith et al., 2000). The MEAQ-30 is a happy compromise. It cuts the original MEAQ by half while maintaining fidelity to the six dimensions of experiential avoidance.

If a valid and reliable shorter form of a long measure is available, researchers have an ethical obligation to use the short form to avoid burdening participants unnecessarily. Further, a difference of roughly 6 min in the administration time of the original MEAQ (which takes about 12 min) and the MEAQ-30 (which takes about 6 min) has significant practical implications. It can mean the difference between not being able to measure experiential avoidance at all and measuring it reliably when only 6 min are available in an otherwise lengthy battery. And when more room is available in a survey, the MEAQ-30, if used instead of the 62-item MEAQ, can help researchers spare about 6 min for another measure of a separate construct of interest, thus expanding the scope of the original study. Most importantly, the MEAQ-30 provides a multidimensional assessment of experiential avoidance. The BEAQ takes about 3 min to complete whereas the MEAQ-30 takes about 6 min. The slight increase in time may well be worth it. It may mean the difference between identifying the right experiential avoidance strategy for a particular population or client to precisely target that strategy in a tailor-made intervention, and missing that level of specificity all together.

## Author contributions

Conceived and designed the study: BKS and JC; Data collection: BKS and JC; Analyzed the data: BKS, JC, PP, and LS; Designed analysis tools: BKS and LS; Wrote the paper: BKS, JC, PP, and LS.

### Conflict of interest statement

The authors declare that the research was conducted in the absence of any commercial or financial relationships that could be construed as a potential conflict of interest.

## Supplementary material

The R data file, Imputation1.Rdata, contains the MEAQ data from one of our imputed dataset. All 25 imputed datasets are available upon request. The text file MEAQitems.txt contains information about the labels of the variables in the data file.
