# Supplementary material for: Using Genetic Algorithms in a Large Nationally Representative American Sample to Abbreviate the Multidimensional Experiential Avoidance Questionnaire
Source: Front Psychol. 2016 Feb 24;7:189. doi: 10.3389/fpsyg.2016.00189 (PMC4764703; doi:10.3389/fpsyg.2016.00189)
Supplement: Supplementary file 2 [file DataSheet2.docx]

# Appendix A

An example R code for running GAabbreviate for scale reduction

The package source (GAabbreviate_1.0.tar.gz) and the reference manual (GAabbreviate.pdf) can be downloaded from the CRAN repository:

<http://cran.r-project.org/web/packages/GAabbreviate/index.html>

The following R code randomly generates data, creates matrices of the 15 items of the measure and the two scales (sub-dimensions or subscales) of the measure (one with 10 item and another with 5), runs a GA with item cost set to 0.01, population size of 50 and maximum number of iterations set to 300, and provides summary and plots of the GA solution.

### Install and load the GAabbreviate package

install.packages("GAabbreviate")

library(GAabbreviate)

### Create a random generated data

nsubject = 100

nitems = 15

set.seed(123)

items = matrix(sample(1:5, nsubject*nitems, replace = TRUE),

nrow = nsubject, ncol = nitems)

scales = cbind(rowSums(items[,1:10]), rowSums(items[,11:15])

### Run GAabbreviate using the items and scales matrices created above

GAA = GAabbreviate(items, scales, itemCost = 0.01, maxItems = 5,

popSize = 50, maxiter = 300, run = 100,

verbose = TRUE)

plot(GAA)

summary(GAA)

GAA$best

GAA$measure

# Appendix B

Conventional criteria for scale abbreviation

Using a method based on confirmatory factor analysis, Marsh et al. (2005) reported the following 6 criteria for selection of items for abbreviating a long form (p. 85):

1. Items that best measured the intended construct as inferred on the basis of corrected item-total correlations (available in most reliability procedures) and the size of standardized factor loadings in CFA.

2. Items that had minimal cross-loadings as evidenced by LISREL’s modification indexes, indicating the extent to which the fit would be improved if an item were allowed to load on a factor other than the one that it was intended to measure and the expected size of the cross-loading.

3. Items that had minimal correlated uniquenesses, particularly with other items in the same scale. In the case where two items within the same scale had substantial correlated uniquenesses, only one of the two items was retained.

4. The number of times that an item was left blank.

5. Subjective evaluations of the content of each item in order to maintain the breadth of content of the original construct.

6. Sufficient items in each scale to maintain a coefficient alpha estimate of reliability of at least .80.

We used these criteria to create and test a short form using the same training (*N*=5913; 75% of the original sample) and testing subsamples (*N*=1971; 25% of the full sample) used for the GA method. The training subset was used to apply the criteria listed above (except point 4, which does not apply to our missing data by design). Instead of LISREL, we used lavaan in R. Some items that met the first criterion (had high corrected item-total correlation and high factor loading) failed to meet the second one (also had high modification index) or third one (had high correlated uniqueness), so we had to manually go through several iterations of the criteria to select items that could meet all criteria as best as possible. A 6-factor CFA of the brief measure using the testing sample yielded a good fit: χ^2^(390) = 402.973, *p* < .001, CFI = .99, TLI = .99, RMSEA = .004, 95% CI [0.00 0.01]. We note that this manual process of item selection was very cumbersome compared to the fully automated process of running the GA. It also relied on our subjective judgment of the selected items, which is arguably problematic because different researchers may have different subjective sense of the suitability of an item. The fit of the short form, MEAQ-30, yielded using the GA method reported in the main text of this paper was comparable (χ^2^(390) = 469.68, *p* < .001, CFI = .97, TLI = .97, RMSEA = .01, 95% CI [0.006 0.01]) to the fit of the measure yielded by the manual method. We believe that the less subjectivity required by the GA method and high efficiency it affords demonstrate its distinctive advantage.
